# A cost function to determine the optimum filter and parameters for stabilising gaze data

**DOI:** 10.16910/jemr.12.2.3

**Published:** 2019-07-04

**Authors:** Pieter Blignaut

**Affiliations:** University of the Free State, South Africa

**Keywords:** Eye-tracking, filters, data quality, accuracy, precision, latency

## Abstract

Prior to delivery of data, eye tracker software may apply filtering to correct for noise. Although filtering produces much better precision of data, it may add to the time it takes for the reporting of gaze data to stabilise after a saccade due to the usage of a sliding window. The effect of various filters and parameter settings on accuracy, precision and filter related latency is examined. A cost function can be used to obtain the optimal parameters (filter, length of window, metric and threshold for removal of samples and removal percentage). It was found that for any of the FIR filters, the standard deviation of samples can be used to remove 95% of samples in the window so than an optimum combination of filter related latency and precision can be obtained. It was also confirmed that for unfiltered data, the shape of noise, signified by *RMS/STD*, is around $\sqrt{2}$ as expected for white noise, whereas lower *RMS/STD* values were observed for all filters

## Introduction

For many, programmers and end users alike, an eye tracker is a black box with its inner workings hidden from its users. The black box consists of both hardware and software. For video-based eye trackers, the hardware may consist of one or more cameras and one or more infrared illuminators. Eye tracker software analyses the video frames and uses features such as images of the pupils and corneal reflections to map to gaze coordinates on a two-dimensional stimulus plane. Programmers connect to an eye tracker through an SDK (Software Development Kit) or API (Application Programming Interface) and utilise the delivered gaze coordinates on specific timestamps. Users have faith that the black box, through its software, delivers high quality data in terms of robustness, accuracy, precision and latency, and they do experiments and reach conclusions that depend on the delivered samples from the black box.

Prior to delivery, the software may apply filtering to correct for noise (unwanted or unknown modifications to the signal) that may originate from either the hardware or the participant. Noise can be attenuated by inspecting neighbouring samples, for example, through the *Kalman* ( 10) or weighted triangular (11) filters. A good overview of filters can be found in Špakov (16).

While filters can be applied to improve precision, there are two major drawbacks. Firstly, it takes control out of programmers' hands, as they might have wanted to implement another filter. The filters mostly depend on parameters such as the window length and/or cut-off thresholds. Manufacturers cannot be sure that a specific filter and/or parameter settings are the best for all scenarios or experimental conditions. Secondly, the filters may induce a latency in the delivery of data, with the effect that data is reported up to 158 ms after an event (e.g. saccade or fixation) occurred (16). Studies where the data is analysed post-hoc, for example usability or market research studies, might not be affected by latency as long as it is consistent. For interactive systems, on the other hand, as in gaming or gaze contingent systems, latencies as small as 50 ms may be crucial.

In this paper, the effect of various filters and parameter settings on accuracy, precision and response time is examined while using a self-built eye tracker that gives the researcher control over the filters and settings. Besides the filtering algorithm, the effect of a dynamic sliding window that changes size based on a dispersion metric and threshold, is also examined.

## Accuracy and Precision

**Figure 1.**
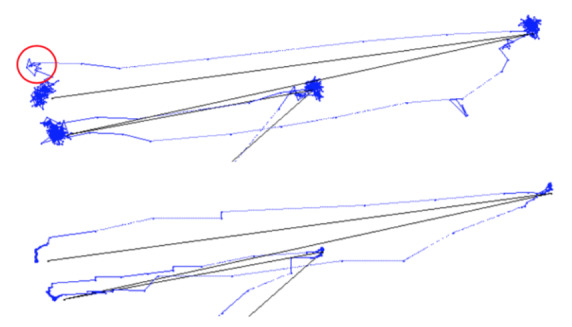
Gaze data of a participant following a target that appears suddenly at random positions. Top: Unfiltered; Bottom: Kalman filter applied over a sliding window of 500 ms.

The spatial accuracy (measured in terms of the absence of systematic error) of an eye tracker is an indication of the extent to which the eye tracker reports the true gaze position accurately. In this paper, when the term "error" is used without other context, it refers to the spatial offset between the actual (true) and reported gaze positions. The Euclidean distance between these two points can be measured in pixels and then converted to degrees of gaze angle that is subtended at the eyes in 3D space. Precision (a.k.a. variable error), on the other hand is defined as the “closeness of agreement between independent test results obtained under stipulated conditions” (ISO, 1994). The precision of an eye tracker refers specifically to the machine's ability to reliably reproduce a measurement (7). In ideal circumstances, if a participant focuses on a specific point on a stimulus plane, successive samples from the eye tracker should report the exact same location. Unfortunately, this is not the case in real life as noise originating from the human participant, the eye tracker hardware and experimental conditions can influence the reporting of gaze data. This has the effect that the reported point of regard (POR) is jittery around the actual POR. With filtering, the POR can be stabilised (*cf* Figure 1).

High precision is needed when measuring small fixational eye movements such as tremor, drift and micro-saccades. Poor precision can be caused by a multitude of technical and participant-specific factors, such as hardware limitations and eye colour (7), and can be detrimental to fixation and saccade detection algorithms.

Several measures of precision can be used (3), but the two most commonly used measures are the root-mean-square ( *RMS*) measure of successive sample-to-sample distances ( *d* in Equation 1) and the standard deviation ( *STD*) obtained from the trace of the covariance matrix of the two dimensions:

(1) $$RMS=\sqrt{\frac{1}{n}\sum_{i=1}^{n}d_{i}^{2}}$$

(2) $$STD=\sqrt{\sigma_{x}^{2}+\sigma_{y}^{2}}$$

Blignaut and Beelders (3) further showed that *RMS* may be dependent on framerate. Holmqvist and Andersson (7) indicated also that these two metrics measure two different characteristics of an eye tracker, namely the noise with respect to the sample-to-sample velocity of the signal and the extent of noise respectively. Refer also to (8) in this regard. These studies proposed the use of a combination of the two metrics to indicate some aspects of the shape of the noise.

(3) $$Shape=\frac{\text{RMS}}{\text{STD}}$$

For white noise (truly random noise), the theoretical value of *RMS/STD* should be $\sqrt{2}$ (5) while filtered data should have a shape value that is less than $\sqrt{2}$. According to Holmqvist and Andersson (7), the ratio of *RMS/STD* depends largely on the filters that are used. It follows that if manufacturers decide on the filter and parameters, *RMS/STD* is eye tracker dependent and therefore it can serve as a “signature” of the eye tracker.

Holmqvist and Andersson (7) further defined an extent measure based on *RMS* and *STD* that quantifies the magnitude of noise as

(4) $$Extent=\sqrt{{\text{RMS}}^{2}+{\text{STD}}^{2}}$$

## Filtering and the effect thereof

Stabilisation of noisy data can be done by applying a finite-impulse response filter on the raw samples in a sliding window that includes historical data up to the latest recorded sample (16). Although filtering produces much better precision of data, especially in terms of *RMS*, it may introduce latencies in the response time due to the usage of a sliding window (16). In other words, better precision can be obtained at the cost of latency.

Linear time-invariant (LTI) filters, such as the Stampe filters ( 18) and the Savitzky-Golay filters (15) have a constant (linear) delay in the output that depends on the number of previous samples that are included (3 and 5 respectively depending on the implementation). For the non-linear time-invariant (NLTI) filters, the sliding window may contain more samples as determined by a parameter.

In this paper, a method is described to remove a certain percentage of samples from the beginning of the sliding window based on a dispersion cut-off threshold. This is similar to the approach of Kumar et al. (11) who used outlier and saccade detectors to manage the number of gaze points in the window, but follows a different procedure.

Filters are characterised by (i) the specific algorithm, (ii) the maximum permissible length of the stabilisation window, (iii) the metric used for dispersion of samples in the window, (iv) the threshold to remove samples and (v) the number of samples to remove.

Figure 1 (top) shows the raw gaze data of a participant following a target as it appears suddenly and in random position on the display area. The sample data appears to be noisy around the target positions. An undershoot with subsequent correcting saccade is encircled.

Figure 1 (bottom) shows the same gaze data after a *Kalman* filter was applied. The sample points during fixations are now aligned linearly in what Blignaut and Beelders (3) termed "ant trails" due to the resemblance of the trails that ants leave in soft sand. Although this filter provides much better precision of data, especially when *RMS* is used, it introduces latencies in the response time (*cf* Figure 6). Unfortunately, it also hides the occurrence of over- and undershoots to some extent.

## Methodology

### Recording of gaze data

A self-built eye tracker with two clusters of 48 infrared LEDs (850 nm), 480 mm apart, and the UI-1550LE camera from IDS Imaging (https://en.ids-imaging.com) was used to capture gaze data at a framerate of 200 Hz. The illuminators (2 × 5 W) were certified by the South African Council for Scientific and Industrial Research as being within the limits set by the COGAIN community (12).

Software was developed using C# with .Net 4.5 along with the camera manufacturer’s software development kit (SDK) to control the camera settings and process the eye video. Data was recorded with a desktop computer with an i7 processor and 16 GB of memory, running Windows 10. A full discussion of the mapping of eye features (pupil centre and corneal reflections) to gaze coordinates and of the calibration process is beyond the scope of this paper. The interested reader can refer to Blignaut (4) in this respect.

Six adult participants were recruited as part of another study that was approved by the ethics committee of the Medical Faculty of the University of the Free State. The participants were presented with a series of 14 targets that appeared at random positions on the display and stayed in position for 1.5s before the next target appeared. Participants were requested to move their gaze to a target immediately when it appears. Although it is possible for a participant to look elsewhere, it was assumed that the target position represents the actual gaze position. Since the aim was not to determine absolute values for the various components of data quality, but to examine the effect of filtering, it was not required to test a larger number of participants.

The raw, unfiltered gaze data was captured and saved, where after several combinations of filters and parameters were applied to calculate the gaze coordinates before they would have been delivered to the user in case of pre-delivery processing. Data for the first and last target were excluded from the analysis due to possible end effects.

### Stabilisation of gaze data through filtering

#### Processing of data

Manufacturers may choose to set the filter and its parameters before a recording commences and the filter is applied prior to delivery of the gaze data to the client. In this experiment, however, every participant’s original unfiltered gaze data was saved. This allowed a post-hoc application and analysis of various combinations of filters and parameter settings.

The filter is applied on the x and y dimensions of the raw samples in a sliding window that includes historical data up to the latest recorded sample (*cf*. Algorithm 1). A queue structure is used as samples are added to the end and removed from the beginning of the queue. The sliding window is capped to a certain maximum number of samples as determined by a parameter. For this experiment, values of 50 ms (10 samples), 100 ms (20 samples) and 500 ms (100 samples) were tested.

1. Add sample to window;

2. **if** *Filter != None* **then**

2.1 Apply filter;

2.2 DeliverGazedata(timestamp, filtered samples);

2.3 **if** *dispersion > threshold* **then**

2.3.1 remove first p % of samples from the window

**end**

**else**

2.4. DeliverGazedata(timestamp, raw samples)

**end**

3. Cap sliding window to maximum length;

**Algorithm 1: Process of stabilisation**

#### Selected filters

The filters and settings in this study were only used to illustrate that various filters and dispersion metrics have an effect on precision and stabilisation time, and that the manner in which precision is interpreted and measured is crucial towards an informed decision about the quality of data that is delivered. Examples of alternative filters include the skewness of sample data in the window, a Gaussian filter ( 1 16), or a bilateral filter (13). The filters that were tested in this study are listed below.

• The *Average* filter returns (average x, average y) of samples in the window.

• The *Triangular* filter applies a larger weight to samples in the middle of the window.

• The *Kumar* filter (11) applies larger weights to the samples at the end of the window.

• A simplified *Kalman* filter as presented in Esmé ( 6), was applied separately on each dimension as indicated below.

*Sort observations in the current dimension*

*list = median fifth of the observations (40th-60^th^ percentile)*

*x = median*

*R = std dev of list //Indication of measurement noise*

P = 10 * R

*for k = 1 to n-1*

*K = P / (P+R) // i.e. initially 10/11*

*x = x + K(list[k] – x)*

P = (1- K) * P

*return x*

• The *Stampe* filters (two filters in succession) were implemented according to Stampe (18). Note that in the algorithm below, the estimated value is not returned, but the values of one or more of the previous elements in the list is updated.

*Stampe1(win)*

*if (n < 3) return;*

*x = win[n-1]; x1 = win[n-2]; x2 = win[n-3];*

*if ((x2 > x1 && x1 < x) || (x2 < x1 && x1 > x))*

*win [n-2] = Abs(x2-x1) < Abs(x1-x) ? x2 : x;*

*Stampe2(win)*

*if (n < 4) return;*

*x=win[n-1]; x1=win[n-2]; x2=win[n-3]; x3=win[n-4];*

*if (x != x1 && x1 == x2 && x2 != x3)*

*win[n-3] = win[n-2] = Abs(x1-x) < Abs(x3-x2) ? x : x3;*

• The Savitzky-Golay filter (15) was implemented with 5 points and corrected convolution coefficients by Steinier, Termonia and Deltour (17):

*for i = 2 to n - 3*

return (-3 * lst[i - 2] + 12 * lst[i - 1] + 17 * lst[i]

+ 12 * lst[i + 1] - 3 * lst[i + 2]) / 35

The *Average*, *Triangular*, *Kumar*, and *Savitzky-Golay* filters can collectively be referred to as finite-impulse response (FIR) filters. For these filters, each point in the history has its own weight when calculating output as a weighted average (16). The *Average*, *Triangular*, *Kumar* and *Kalman* filters are NLTI filters.

#### Removal of samples

Samples in the sliding window that belong to a previous fixation or are part of the incoming saccade will cause both instability and latency in the reported POR. Except for the Stampe and Savitzky-Golay filters, a certain percentage of samples are removed from the beginning of the sliding window if the samples do not conform to a certain threshold regarding their dispersion (Step 2.3 in Algorithm 1).

Note that at 200 Hz, a 100 ms window contains 20 samples and 95% removal means removing all but 1 sample. For a 500 ms window (100 samples), 95% removal means that only the last 5 samples are retained. Effectively, this means that when the threshold of dispersion is exceeded, the history buffer is largely wiped.

The dispersion metric, threshold for dispersion and percentage of samples to remove, are specified as parameters. For this study, thresholds in (0.05°, 0.1°, 0.5°, 1.0°, 2.0°) and percentages of 5, 50 and 95 were tested. These parameters were selected to be representative of the range of possibilities as identified by Blignaut (2).

Four different metrics were used in turn to measure dispersion. The interested reader can refer to Salvucci and Goldberg (14) and Blignaut ( 2) for a discussion of these metrics.

• *Sample-to-sample (S2S)*: The maximum distance between successive samples. Since the eye tracker samples points at a constant rate, this can also be interpreted as a velocity measure.

• *Radius*: The largest distance from the centre to any sample in the window.

• *Max-Min*: The maximum horizontal and vertical distance covered by the samples. For this study, it was defined as *((Max X - Min X) + (Max Y - Min Y ))/2*, which denotes the average of the horizontal and vertical dispersion.

• *STD*: The precision of samples in the window according to Equation 2.

#### Summary of the stabilisation process

In summary, 735 combinations of filters and parameters were tested:

3 filters (No filter applied, Stampe, Savitzky-Golay)

(No stabilisation window)

+ 4 filters (Kalman, Average, Triangular, Kumar)

× 3 stabilisation windows (50 ms, 100 ms, 500 ms)

(No removal of samples)

+ 4 filters (Kalman, Average, Triangular, Kumar)

× 3 stabilisation windows (50 ms, 100 ms, 500 ms)

× 4 dispersion metrics (STD, S2S, Radius, Max-Min)

× 5 thresholds for removal (0.05°, 0.1°, 0.5°, 1°, 2°)

× 3 removal percentages (5%, 50%, 95%)

### Data analysis

**Figure 2.**
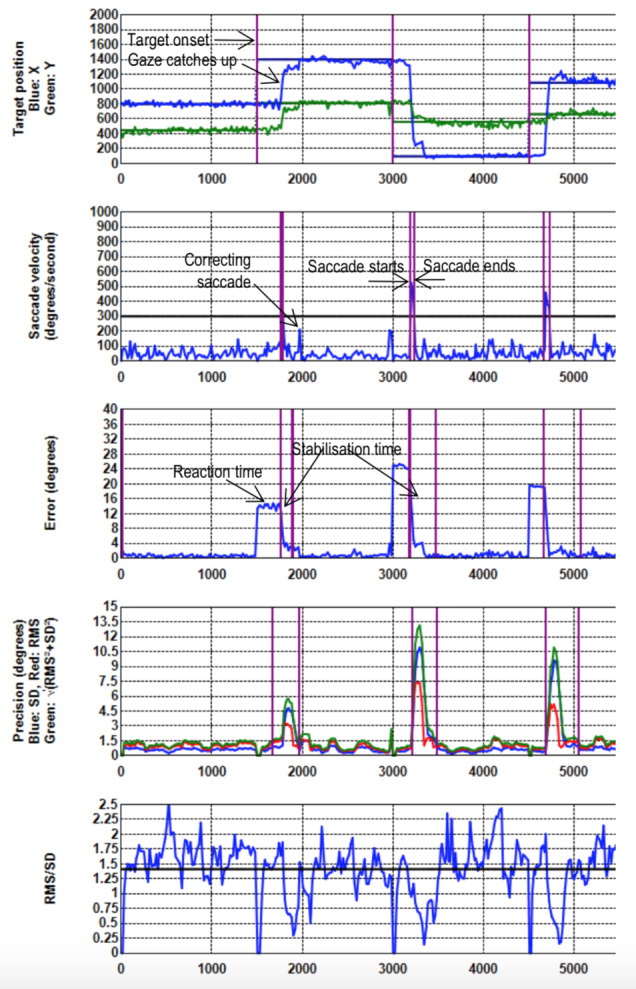
Target position, Saccade velocity, Error, Precision (*RMS*, *STD*, *Extent*) and *Shape* against *Time* (ms) for the first three targets of a specific recording. No filtering is applied. During periods of stable gaze, the shape values are around $\sqrt{2}$.

The period from onset of one target to the next can be divided in four phases (Table 1). Graphs of specific measures of data quality for a single recording are shown in Figure 2. The vertical purple lines indicate the start and the end of the respective phases.

**Table 1:**
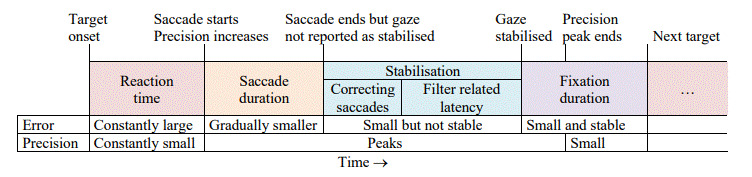
Timeline showing phases of data processing during target presentation

• *Reaction time* is the time from target onset to the start of a saccade. The time taken by the participant to start a response is participant specific and is not considered in this paper.

• *Saccade*: Participants make a quick saccade to position their gaze on the new target. The duration and speed of saccades depend on the distance from one target to the next, as well as on participant characteristics, and are not of interest for the current study. Saccades were identified by a threshold of 300 deg/s. Although this is higher than normal, the nature of the stimulus was such that participants had to make long fast saccades towards the next target. This high value ensured that a clear distinction could be made between saccades and noisy data. A second saccade is possible to correct for overshoots and undershoots.

• *Stabilisation time* is expressed in terms of the time from the end of an initial saccade (when the actual gaze is on the target) until the distance between the target and the reported POR stabilises (*cf* Figure 2) (reported gaze is stable on the target). This is attributed to initial undershoots and overshoots after a saccade plus the *latency* induced by the specific filter (referred to as *fLatency*). The end of this period is marked when the absolute difference between the current offset (see below) and the offset five samples later is less than 0.2°.

For example, the last saccade in Figure 2 starts at 4659 ms and ends at 4727 ms. The gaze is stabilised at 4886 ms. This gives a stabilisation time of 159 ms.

• *Fixation*: The period during which the reported gaze is stable on a target. This period ends when the target moves to its next position. The accuracy and precision that can be achieved with a specific filter and parameter settings were calculated during this period.

Three dependent variables were calculated for each filter/parameter combination and averaged over all participants and targets, namely filter related latency, average error and precision. These agree with the comparison criteria of Špakov (16) of delay, closeness to the idealized signal and smoothness respectively, but are measured differently.

• Filter related *latency* forms part of stabilisation time after a saccade.

• The *average error* (spatial offset) with respect to the target during the fixation phase. It is important to note that this paper is not about the absolute accuracy that can be obtained with the eye tracker, but about the difference in error between the filtered and unfiltered cases. In other words, the question is asked whether filtering has an effect on the magnitude of the spatial offsets between actual and reported gaze positions.

The average error during the fixation phase was regarded to be representative of what can be achieved with the specific filter and parameter settings.

• *Precision* of delivered gaze coordinates in terms of RMS (Eq 1), *STD* (Eq 2), *Shape* (Eq 3) and *Extent* (Eq 4). Precision cannot be calculated on individual samples. For this study, precision was calculated based on a sliding window of 100 ms.

- At the onset of a saccade, precision increases and peaks when a saccade is at its fastest. The precision then decreases until the reported gaze is stable on the target (*cf* Figure 2). Because precision is calculated with a sliding window, the precision peak does not stabilise immediately when the gaze comes to rest after a saccade.

- The average precision during the fixation phase was regarded to be representative of what can be achieved with the specific filter and parameter settings.

The complete analysis procedure can be summarised as in Algorithm 2.

**for** *No filter* **do**

Calculate dependent variables

**foreach** *filter* **do**

**foreach** window size **do**

**for** No dispersion metric applied **do**

Calculate dependent variables

**foreach** dispersion metric **do**

**foreach** threshold **do**

**foreach** removal percentage **do**

Calculate dependent variables

**Algorithm 2: Analysis procedure**

### Cost function

In order to find the optimum combination of parameters, a cost function, *C*, was defined. If we want to minimise the extent of precision, ($E=\sqrt{{\text{STD}}^{2}+{\text{RMS}}^{2}}$) and filter related latency (*L*), we can normalize the aggregated gaze data of every target point in terms of the z-scores ( *E_n_* and *L_n_*).

(5) $$C=\frac{w_{L}L_{n}+w_{E}E_{n}}{w_{L}+w_{E}}$$

where *w_L_* and *w_E_* are the weights for stabilisation time and extent of precision respectively. If, for example, limited latency is preferable over good precision, we can define

(6) $$C=\frac{3L_{n}+E_{n}}{4}$$

Now we find the parameter set (filter, window length, dispersion metric, threshold and removal percentage) for the minimum value of *C*, averaged over all participant recordings and target points.

## Results

### Graphical comparison of the effects of filtering

Figure 2 shows graphs for target position, saccade velocity, error, precision (*RMS*, *STD* & *Extent* on one graph) and *Shape* for the first three targets for one participant without any filtering. In the top graph, the straight lines indicate the actual target position in pixels while the wavy lines indicate the position as reported by the eye tracker. The delay in participant response after onset of a new target can be seen and this agrees with the periods of large offsets in the third graph. Note the correcting saccade on the target at 1,971 ms after an over-shoot (second graph) that agrees with the temporary gaze stabilisation in the first graph.

**Figure 3.**
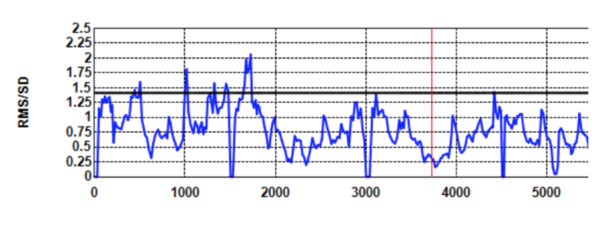
*Shape* against *Time* (ms) for the same recording with Kalman filtering (window = 500 ms) and no sample removal. The lower shape values are indicative of filtering

Without filtering, the shape of precision (*RMS/STD*) is around $\sqrt{2}$ (dark horizontal line on the fifth graph in Figure 2) during periods of stable gaze which is indicative of white noise. Figure 3 shows the *Shape* values of the same recording when a *Kalman* filter with 500 ms sample window is applied without dispersion cut-off. The lower values are indicative of the application of a filter.

**Figure 4.**
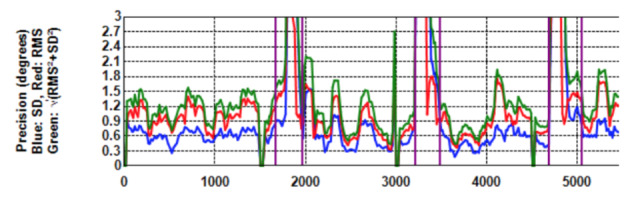
Precision (RMS (Red), STD (Blue), Extent (Green)) with no filtering. This is the same as Figure 2 but the scale is larger. Note that the red graph runs above the blue one (RMS > STD).

**Figure 5.**
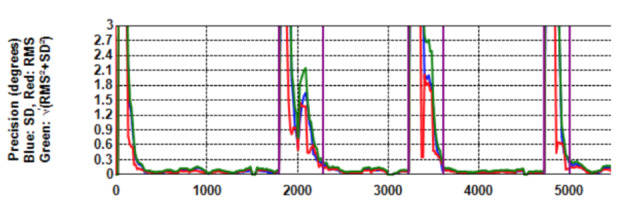
Precision with Kalman filtering (window = 500 ms) and dispersion cut-off (metric STD, threshold=1.0°, 95% removal) on a larger scale. In comparison with Figure 4, the effect of filtering is clear. Now, RMS < STD. The effect of the correcting saccade is also visible.

Figure 4 is the same as Figure 2 (fourth), but on a larger scale. When no filtering is applied, RMS (the red curve) is constantly greater than *STD* (blue). With filtering (Figure 5), both *RMS* and *STD* are reduced, but *RMS* more so with the effect that precision extent (green curve) runs more or less on top of *STD*.

### Error and other variables without filtering

Table 2 shows the values of the various dependent variables when no filtering is applied, averaged over participant recordings and target points. These values can serve as a frame of reference in the subsequent discussion.

The average latency of 56 ms can probably be attributed to undershoots and overshoots. These are natural and cause short correcting saccades, which take time before gaze stabilises. This value for latency when no filter is applied can be regarded as an offset for the current data set. The difference between this value and the latency when the respective filters are applied is regarded as the actual filter-related latency.

**Table 2:** Values of dependent variables when no filter is applied (n = 67).

**Variable**	**Mean**	**STD**
Error	1.25°	1.87°
RMS	0.63°	0.42°
STD	0.47°	0.43°
Shape	1.43	0.29
Extent	0.80°	0.59°
S-time	56.0 ms	60.6 ms

### Effect of filtering without dispersion cut-off

**Figure 6.**
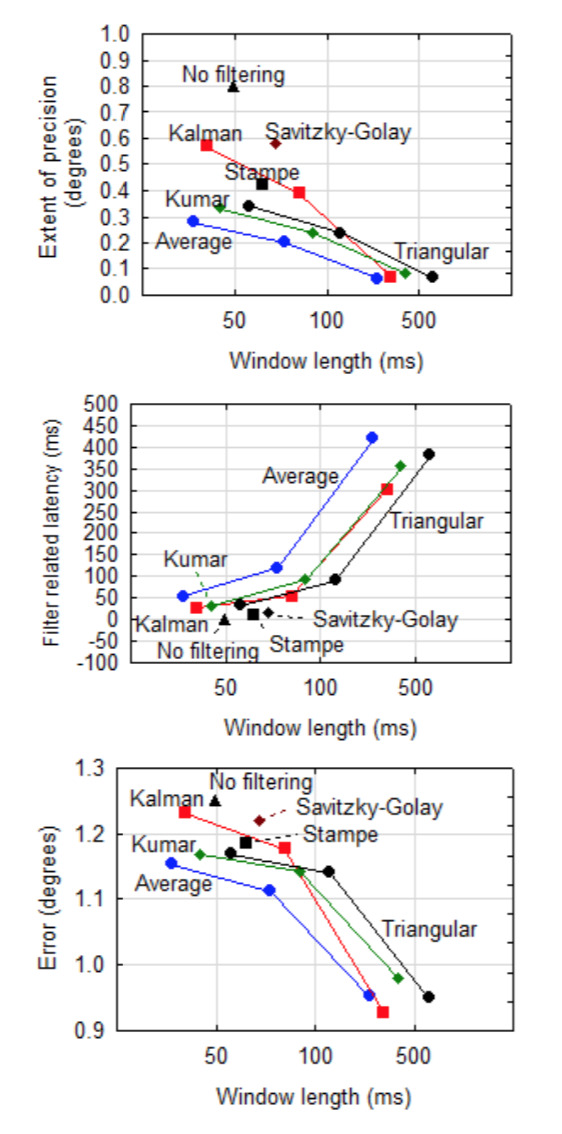
Precision, filter related latency and error per filter against length of the stabilisation window when no dispersion cut-off is done. (Note: Although the window lengths are exactly 50 ms, 100 ms and 500 ms, the data points are horizontally offset a bit to reduce clutter and separate the lines.)

Figure 6 shows extent of precision, filter related latency and error (Euclidean distance between actual and reported point of regard) against length of the stabilisation window when no dispersion cut-off is done and the entire window of samples is used for calculations. Any one of the NLTI filters provided a significantly (α=0.001) better precision at the cost of a longer stabilisation time in comparison with the raw data (no filtering). The differences with the raw data are larger when more samples (longer window) are used.

It is important to note that the filter related latency increases as precision improves. In other words, better precision can be obtained at the cost of latency. Although not always significant (α=0.05), the *Average* filter gives the best precision but the worst latency. The *Kalman* filter is less effective at a shorter stabilisation window, but appears to be good for longer windows. For the other filters, the effect of a longer window on precision, although significant (α=0.001), is not extreme and it may be beneficial to sacrifice a bit of precision for the sake of better response.

As expected, the latencies of the Stampe and Savitzky-Golay filters are short, but their precision values are worse than that of the other filters.

Although it might seem that the error decreases with a longer window for NLTI filters, this deception is the result of the scaling in Figure 6 (bottom). Neither the interaction (*F(6,829) = 0.158, p > .999*) nor the individual effect sizes of *Filter* ( *F(3,829) = 0.017, p = .997*) and *Window* ( *F(2,829)= 1.22, p = .294*) were significant contributors to the magnitude of the error.

### The effect of dispersion cut-off

**Figure 7.**
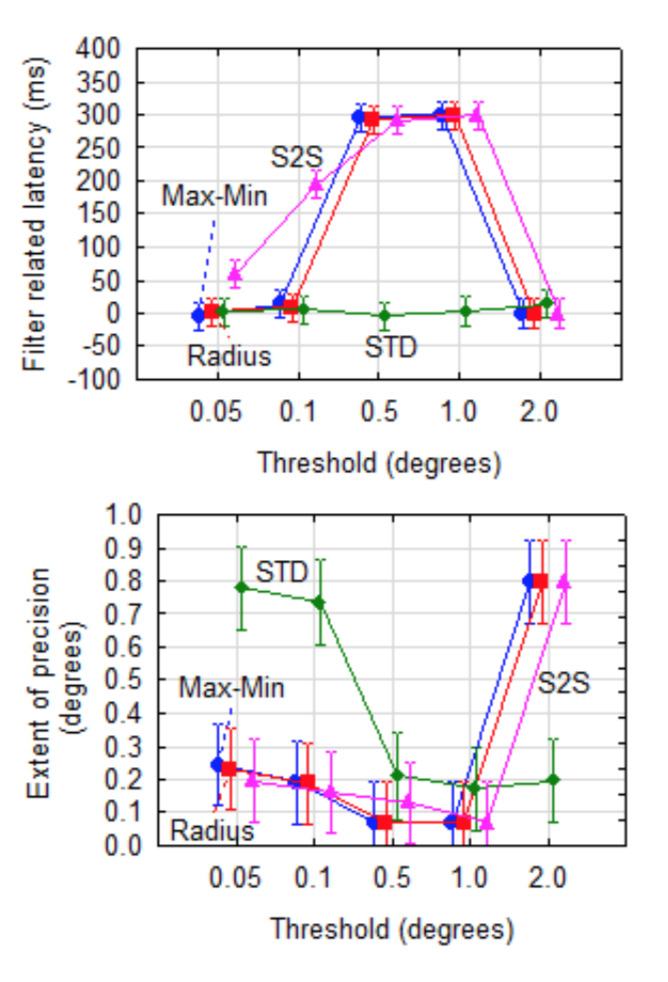
Filter related latency and extent of precision for combinations of dispersion metric and cut-off threshold for the Kalman filter (window = 500 ms, removal = 95%). The vertical bars indicate 95% confidence intervals.

As an example of the effect of dispersion metric and cut-off threshold, Figure 7 shows the extent of precision and filter related latency for the Kalman filter using a stabilisation window of 500 ms and removing 95% of samples based on different combinations of dispersion metric and threshold.

For latency, the interaction effect of these two parameters was significant (F(12,1331)=61.4, p<0.001). Using Tukey's post-hoc test, it was determined that the STD dispersion metric was significantly (α=0.001) better (shorter latency) than any of the other metrics for thresholds of 0.5° and 1.0°.

For precision, the interaction of dispersion metric and threshold was also significant (F(12,1331) = 13.7, p<0.001). The STD metric proved to be worse (higher) than the other three for lower thresholds. With *w_L_=3* and *w_E_=1,* the STD metric at a threshold of 0.5° provided the best combination of latency (0 ms) and extent of precision (0.21°).

### Using a cost function to determine the optimum parameter set

**Figure 8.**
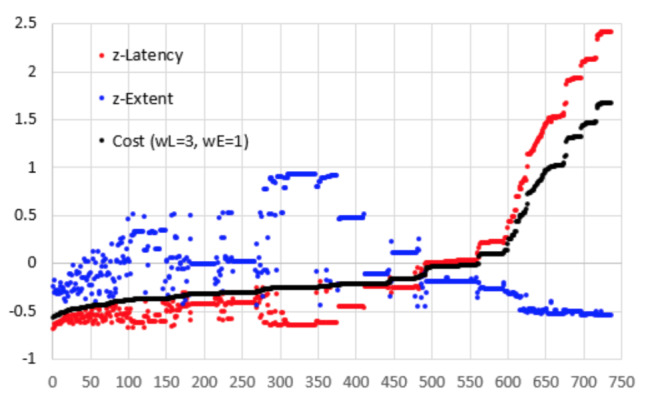
Cost (*w_L_=3, w_E_=1*) for 735 combinations of filter and metrics.

Figure 8 shows a graph of cost (*w_L_=3, w_E_=1*) against the 735 possible combinations of filters and metrics. The graph also shows the normalised latency and normalised extent of precision.

The cost line rises slowly in the beginning and there are 80 combinations with a cost of -0.40 or less and 295 combinations with a cost of -0.25 or less. It is only for the last 177 combinations that the line rises sharply.

**Figure 9.**
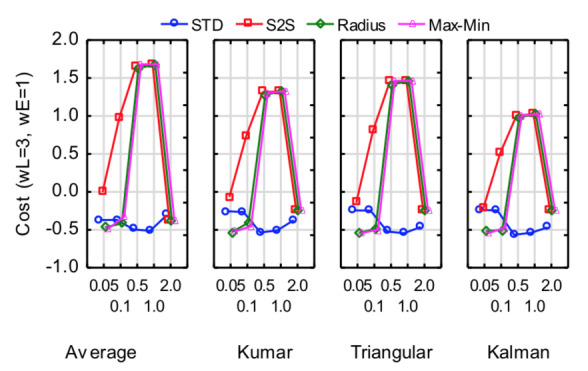
Cost against removal metric and threshold for the NLTI filters for window = 500 ms and 95% sample removal.

Combinations with a cost of -0.5 or below along with some other specific combinations are listed in Table 3. The worst cost value is 1.68 with a corresponding latency of 420 ms (normalised 2.418). Amongst the good performers, the only constant variables are 500 ms for the stabilisation window with 95% (19 of the 20) of samples removed. Using these values, Figure 9 shows graphs of the cost against metric and threshold for the four NLTI filters. Although some of the metrics deliver slightly lower cost for some of the filters, it seems as though the STD metric at a threshold of 0.5° or 1.0° provides consistently better results. Furthermore, there is no significant difference (α=0.05) between the four filters at these values.

**Table 3:** Combinations of filters and metrics with a cost (*w_L_=3, w_E_=1*) of -0.5 or less along with values for no filtering and the Stampe and Savitzky-Golay filters.

	Filter	Win (ms)	Dispersion	fLat (ms)	Error (deg)	Precision	Cost				
			Metric	Thr	Rem			RMS	SD	Extent	
1	Kalman	500	STD	0.5°	95%	0	1.21	0.13	0.16	0.21	-0.565
2	Kalman	500	M-M	0.05°	95%	0	1.20	0.14	0.19	0.25	-0.546
3	Tri	500	STD	1.0°	95%	2.0	1.14	0.10	0.15	0.18	-0.545
4	Tri	500	M-M	0.05°	95%	0.3	1.14	0.11	0.16	0.19	-0.545
5	Tri	500	Rad	0.05°	95%	2.0	1.16	0.10	0.15	0.18	-0.542
6	Kalman	500	STD	1.0°	95%	3.2	1.13	0.10	0.14	0.17	-0.540
7	Kumar	500	STD	0.5°	95%	2.1	1.20	0.12	0.15	0.19	-0.535
8	Kumar	500	Rad	0.05°	95%	3.9	1.12	0.11	0.15	0.18	-0.531
9	Kumar	500	M-M	0.05°	95%	5.8	1.14	0.10	0.14	0.17	-0.525
10	Tri	500	STD	0.5°	95%	3.1	1.21	0.13	0.16	0.21	-0.522
11	Kalman	500	Rad	0.05°	95%	2.1	1.20	0.13	0.19	0.23	-0.517
12	Kumar	500	STD	0.5°	50%	7.6	1.23	0.07	0.16	0.18	-0.514
13	Average	500	STD	1.0°	95%	5.0	1.17	0.10	0.18	0.21	-0.514
14	Tri	500	STD	0.5	50%	6.5	1.25	0.09	0.17	0.19	-0.512
15	Kumar	500	STD	1.0	95%	10.9	1.17	0.08	0.13	0.15	-0.509
16	Kalman	500	Rad	0.1	95%	8.2	1.13	0.10	0.16	0.19	-0.505
…											
125	Stampe					11.2	1.19°	0.26°	0.33°	0.42°	-0.372
…											
275	Savitzky-Golay			13.6	1.22°	0.40°	0.40°	0.58°	-0.281		
…											
334	No filter					0	1.25°	0.63°	0.48°	0.80°	-0.245

## Summary and Conclusions

### Shape of precision

The proposal by Holmqvist, Zemblys and Beelders (8) to describe the shape of eye tracking noise as the ratio of the commonly used measures of RMS and STD, was shown to be effective to indicate the effect of filtering. When no filtering is applied, *RMS* is consistently greater than *STD*. With filtering, both *RMS* and *STD* are reduced, but *RMS* more so with the effect that *RMS* is now consistently less than *STD*. Shape was confirmed to be around $\sqrt{2}$ when no filtering was applied (the theoretical value for Gaussian distributed noise) and below $\sqrt{2}$ with some filtering. This provides, therefore, an easy measure to determine if manufacturers provide filtered data to their end users as even the LTI filters with short latencies, such as Stampe (18), provide shape values less than $\sqrt{2}$.

### Effect of filters

Although it is known that filtering can cause latencies (16), this paper attempted to visualise and quantify these effects and also compare different filters. We also proposed a procedure whereby a percentage of samples are removed from the beginning of a window depending on a dispersion metric and threshold.

It was confirmed that when no dispersion cut-off is done and the entire window of samples is used for calculations, any one of the filters tested in this study provided a significantly better precision at the cost of significantly longer stabilisation time in comparison with the raw data. The *Kalman* filter does not perform well when a short window is used.

Utilising a dispersion metric along with a cut-off threshold assists towards reducing the sliding window to minimise the latency during saccades. The sliding window will return to its normal length when gaze is stable - thereby ensuring better precision.

Using the *Kalman* filter with a stabilisation window of 500 ms and 95% samples removed as example, it was shown that different values for the threshold have a significant effect on filter related latency stabilisation time and precision for the *Max-Min,* *S2S* and *Radius* metrics (cf Figure 7). The STD metric did not affect filter related latency significantly (α=.001) and was consistently low. Precision was significantly (α=.001) worse for thresholds of 0.05° and 0.1° than for higher thresholds. This means that STD proved to be the best metric to use as long as higher thresholds are applied.

Neither the interaction, nor the individual effect sizes of the filter that is used or the window length contributed significantly (α=.05) to the accuracy that can be obtained with the specific eye tracker.

### Finding an optimum filter

A cost function (Equation 5) was defined in order to find the optimum combination of parameters to select a filter with the best compromise of precision and latency.

Many of the combinations of filter, window length, dispersion metric, threshold and percentage of removal delivered a low cost value, but about 24% of the combinations performed really badly in terms of latency and precision. A window size of 500 ms and removal percentage of 95% proved to be consistently present amongst the best performing combinations. The STD metric for dispersion at thresholds of 0.5° and 1° proved to deliver consistent low cost values for all non-linear filters.

There were no significant (α=.05) differences between the cost values for the four non-linear filters at these values although the *Kalman* filter with a window of 500 ms in combination with the STD metric for dispersion (threshold 0.5°, 95% of samples removed) was the best performer for *w_L_=3 and w_E_=1*. This filter produced an improved precision over the unfiltered value at the cost of no extra stabilisation time. This is different from the finding of Špakov (16) who found this filter to be unacceptable and might be due to (i) the specific implementation thereof (see the algorithm above and (ii) the dispersion metric and threshold that was used to reduce the size of the window of samples.

Although the filter related latencies of the Stampe and Savitzky-Golay filters are also very short (11.2 ms and 13.6 ms respectively), the resulting precision values (0.42° and 0.58°) are much higher than that of the non-linear filters. This agrees with the results of Špakov (16) that these filters have "poor smoothness".

In summary, it can be concluded that a 500 ms stabilisation window along with a removal of 95% of samples based on the STD metric at 0.5° or 1° threshold is likely to produce very good results irrespective of the NLTI filter that is applied.

## Limitations and future research

The approach of a dynamic sliding window that changes size depending on a dispersion metric and threshold might not work for smooth pursuit eye movements as there is no clear distinction between saccades and fixations. Future research should investigate this.

This research was done with a self-built low-cost eye tracker and all offset and precision values should be regarded as specific to this tracker. It might be insightful to repeat the work with a high-end commercial eye tracker.

It was concluded above that neither filter, nor window length has a significant effect on the spatial accuracy of the eye tracker. However, the trend as observed in Figure 6 (bottom), indicates that it might be worthwhile to also include accuracy in the cost function.

In this study, some discrete values for window length (50 ms, 100 ms, 500 ms), removal thresholds (0.05°, 0.1°, 0.5°, 1° and 2°) and removal percentages (5%, 50%, 95%) were used. The study could be repeated with other values or a continuum of values in an interval based on the findings above.

The algorithm that was used to implement the Kalman filter, was based on the median fifth of observations after the data was sorted by position. It is possible that smaller or larger windows can affect the results.
